# Announcing Computational and Structural Biotechnology Journal: not just another open-access journal

**DOI:** 10.5936/csbj.201204001

**Published:** 2012-02-10

**Authors:** Gianni Panagiotou

**Affiliations:** a Center for Biological Sequence Analysis, DTU-Systems Biology, Building 208, Technical University of Denmark, DK-2800 Kgs. Lyngby, Denmark; b Novo Nordisk Foundation Center for Biosustainability, Metagenomic Systems Biology, Technical University of Denmark, Fremtidsvej 3, 2970 Hørsholm, Denmark

We are pleased to announce the launch of *Computational and Structural Biotechnology Journal* (*CSBJ*), which will publish its first issue in April 2012. *CSBJ* aims to become a significant international participant in the interface of biology and computer science and publish high quality research across the entire biotechnology spectrum. A literature survey shows that the reliance upon computational methods has been growing steadily over the past three decades across all disciplines of biology. As the amount of available data grows in various biotechnology fields, the need for computational approaches to manage, analyze, predict and model grows as well. Furthermore, as technology advances, the focus of computational biology research expands to meet the new challenges and co-evolve with them. We have high expectations that *CSBJ*, by hosting high-quality research and review papers on modern biotechnology, will become a specialized forum and provide opportunities for communication among the scientists of the world.

The scope of *CSBJ* is wide and ambitious; that's why we placed high-priority on ensuring the participation of well-known experts from around the globe in the Editorial team. The Editorial team consists of up- and –coming scientists, who are the new leaders in their respective fields, to ensure a smooth progression from the journal's launch to recognized status as a fully established publishing platform on all aspects of computational and structural biotechnology. There are currently more than 50-leading scientists in the Editorial board from top-quality universities as *Harvard, Yale, ETH, DTU, Max Plank Institute, Columbia University, California Institute of Technology, University of Oxford* and many other prestigious institutions. The Editor-in-Chief, the Associate Editors and the Editorial Board will work synergistically for fair, swift and transparent peer review process to publish timely and high-quality papers.

To make a decision whether a manuscript fits the format and standards of *CSBJ* the Editor-in-Chief or corresponding specialists from the Editorial Board will immediately screen a submission. Appropriate manuscripts will be sent for peer review to at least two experts, who are expected to provide an independent assessment of suitability for publication in *CSBJ*. Based on their reports and scoring, a final decision will be made regarding acceptance of the manuscript. The Editor-in-Chief reserves the right to make final decisions concerning each manuscript submitted to the journal within the shortest possible period. We will aim to make editorial decisions in 6 weeks from the date the article was submitted through the journal submission system, generally with only one round of revisions. However, we encourage our Editorial Board members as well as our reviewers to be motivated critical advisers for improving a manuscript.

*CSBJ* is not of course the first journal that by adapting the open-access model enables free and universal access resulting in dissemination to the widest possible audience. The advantages of the open access-publishing model, and especially the correlation between open-access with higher downloads and higher citations leading to higher Impact Factor, has already been suggested. It is also true that with a free and universal online access, the financial or grant status of the researcher will not influence his/her ability to access articles. At first sight the model of *CSBJ* does not significantly differ from that proposed by other open-access journals, however, in reality *CSBJ* is not just another open-access journal; the high publication fee that most of the journals request is excluding all the resource-poor countries. *CSBJ* wants scientists from developing countries not only as readers of the material that their counterparts in resource-rich countries publish, but as active participants in disseminating research findings. Therefore, for all articles accepted for publication a minimum article-processing charge will be levied to cover the costs incurred by open access publication.

We invite you to work together with us to create this new and high-visibility open-access forum for modern biotechnology. We look forward to receiving your submissions for consideration in the second issue of *CSBJ*.

